# Supplementary material for: Technology-assisted white cane: evaluation and future directions
Source: PeerJ. 2018 Dec 10;6:e6058. doi: 10.7717/peerj.6058 (PMC6292384; doi:10.7717/peerj.6058)
Supplement: Supplemental Information 1 [file peerj-06-6058-s001.doc]

| **Section/topic** | **#** | **Checklist item** | **Reported on page #** |
| --- | --- | --- | --- |
| **TITLE** | | |  |
| Title | 1 | Identify the report as a systematic review, meta-analysis, or both.  **Answer:** Systematic Review, more preferably a review article |  |
| **ABSTRACT** | | |  |
| Structured summary | 2 | Provide a structured summary including, as applicable: background; objectives; data sources; study eligibility criteria, participants, and interventions; study appraisal and synthesis methods; results; limitations; conclusions and implications of key findings; systematic review registration number.  **Structured Abstract:**  **Background.** Several technology-assisted aids are available to help blind and visually impaired people perform their daily activities. The current research uses the state-of-the-art technology to extend traditional navigational aids to produce solutions that are more reliable. In this regard, a white cane is no exception, which is extended using the existing technologies to design Electronic Travel Aids (ETAs), Electronic Orientation Aids (EOAs), and Position Locator Devices (PLDs). Although several review articles uncover the strengths and limitations of research contributions that extend traditional navigational aids, we find no review article that covers research contributions on technology-assisted white cane. The authors attempt to fill this literature gap by reviewing the most relevant research articles published during 2010-2017 with the common objective of extending white cane with the existing technology.  **Methods.** The authors have collected the relevant literature published during 2010-17 by searching and browsing all the major digital libraries and publishers’ websites. The inclusion/exclusion criteria were applied to select the research articles that are relevant to the topic of this review article, and all other irrelevant papers were excluded. Among the 573 (530 through database search and 43 through other sources) initially screened papers, the authors collected 224 full-text articles, which after applying exclusion/ inclusion criteria resulted in 32 papers to be included in the evaluation, comparison, and discussion.  **Results.** The findings show that the research trend is shifting towards developing a technology-assisted white cane solution that is applicable in both indoor and outdoor environments to aid blind users in navigation. In this regard, exploiting smartphone to develop low-cost and user-friendly navigation solution is among the best research opportunities to explore. In addition, the authors contribute a theoretical evaluation framework to compare and evaluate the state-of-the-art solutions, identify research trends and future directions.  **Discussion.** Researchers have been in the quest to find out ways of extending white cane existing technology. However, for a more reliable extension, the design should be user-centric characteristics. It should be portable, reliable, trust-worthy, lightweight, less costly, less power hungry, and require minimal training. In this regard, smartphones, which are the ubiquitous and general-purpose portable devices, should be considered to exploit its capabilities in making technology-assisted white cane smarter and reliable. | 1 |
| **INTRODUCTION** | | |  |
| Rationale | 3 | Describe the rationale for the review in the context of what is already known.  **Answer:**  Blind people are an integral part of our society, who have equal rights of enjoying the beauty of life. However, the lack of vision keeps them limited to a specific restricted set of activities and for others they rely heavily on fellow human beings. Among such to these barriers, one inevitable issue is the navigation in the surroundings to accomplish different tasks. For this purpose, they used traditional navigation aids included guide dog, fellow human and white cane, in which the former two are expensive while the latter is limited to certain basic functionalities.  With the inception and proliferation of ubiquitous sensing and computational technology, researchers have been in the quest of finding out ways to extend the basic functionalities of white cane to enable blind people perform most of these navigation-related activities independently. In this regard, what has been accomplished so far and what are yet to be achieved, are potential research questions that need immediate inquiry. The paper aims to fill this literature gap, as to the best of our knowledge, so far no such review has been contributed by the research community. To answer how the paper is different from the available review and survey articles, the relevant except from the manuscript is included below:  “Several state-of-the-art review articles (Fallah et al. 2013; Tapu et al. 2014) cover research and application technologies related to white cane. (Fallah et al. 2013) present a comprehensive review of indoor navigation systems and highlight the techniques used in path planning, user localization, environment presentation and user-system interaction. (Tapu et al. 2014) survey the wearable systems used by blind users in outdoor environments and identified advantages and limitations of each of the studied system. Several performance parameters were introduced to classify the reported systems by giving qualitative and quantitative measures of evaluation. (Kim et al. 2016) conducted a user study on the characteristics of long cane that the researchers may consider potentially in developing ETAs. They focused on the orientation and sweeping of long cane while it is in constant contact with the ground. They measured the average cane sweeping angle, tilt angle, and grip rotation deviation of a long cane. Besides minor variation among users, for about 90% of the subjects, the index finger and thumb are in contact with cane handle. Also, they found significant differences among the subjects regarding sweeping range with low variations in sweeping frequency.  Besides their significance and valuable research implications, the scope of these survey and review is limited only to the technologies used for navigation in indoor and outdoor environments and their pros and cons including, e.g., device power consumption, intrusiveness, user acceptability, etc. Also, these studies are generic covering some general aspects including, e.g., locating the user in the environment, path-planning techniques, user-system interaction, device operation, sensor information translation, etc., and unable to cover research articles that have extended white cane with technology to develop low-cost and user-friendly navigation solutions. The only exception is (Kim et al. 2016). However, it is a user study on long cane sweeping angle, tilt angle, and grip rotation. Also, due to its scope, it is unable to cover other important factors that are mandatory for the design of suitable ETAs.” | 2-3 |
| Objectives | 4 | Provide an explicit statement of questions being addressed with reference to participants, interventions, comparisons, outcomes, and study design (PICOS).  **Answer:**  The authors aim to investigate “How the existing sensing and computational technology has been exploited in extending white cane,” and “whether these solutions are effective enough to fulfill the needs of blind users.” The relevant except from the manuscript is included below:  “This review article fills this literature gap to understand the potential role of white cane in developing user-friendly navigation aids. The paper evaluates the major aspects of navigation aids including operating environment, device sensing range, computational device, information presentation techniques, etc., which can be exploited in developing more efficient and effective technology-assisted white cane. The paper also identifies the current trends in the relevant literature published during 2010-17, identifies trends and contributes future research directions. More specifically, the contributions of the paper include:   - To identify the state-of-the-art in technology-assisted white cane for obstacle detection, navigation, and orientation by reviewing relevant research publications published during 2010-2017. - To contribute a theoretical evaluation framework for comparing the existing technology-assisted white cane solutions, identify current research trends, and possible future directions.” | 3 |
| **METHODS** | | |  |
| Protocol and registration | 5 | Indicate if a review protocol exists, if and where it can be accessed (e.g., Web address), and, if available, provide registration information including registration number.  **Answer:**  Not Applicable |  |
| Eligibility criteria | 6 | Specify study characteristics (e.g., PICOS, length of follow-up) and report characteristics (e.g., years considered, language, publication status) used as criteria for eligibility, giving rationale.  **Answer:**  The authors collected relevant conference and journal papers of high academic significance, published mostly during 2010-17. The articles written in English language were selected. |  |
| Information sources | 7 | Describe all information sources (e.g., databases with dates of coverage, contact with study authors to identify additional studies) in the search and date last searched.  **Answer:**  **Databases:** Google Scholar, Springer, ScienceDirect, ACM digital library, and IEEE  **Publication year**: 2010-17  **Last date of performing search**: January 25, 2018 | 3 |
| Search | 8 | Present full electronic search strategy for at least one database, including any limits used, such that it could be repeated.  **Answer:**  The authors mostly used Google Scholar using keywords: white cane + virtual white cane + technology-assisted white cane + assistive tools for visually impaired and handicapped + blind assistive technologies + navigation aids for blind people, etc. The publication year range was set to 2010-2017. By manual screening, the authors selected potentially relevant and related paper. The relevant identified paper was then selected to go to its hosting website, where its title, abstract, and academic significance were inspected, and downloaded it if found related/relevant to the topic/theme of this paper. The authors also use the “Cited by” option in Google scholar, to identify potentially relevant and latest papers. Finally, the References list of each paper was inspected to see if there is any relevant paper to be included. | 4 |
| Study selection | 9 | State the process for selecting studies (i.e., screening, eligibility, included in systematic review, and, if applicable, included in the meta-analysis).  **Answer:**  Eligibility/Inclusion Criteria:   - Research papers that extend white cane with the available assistive technologies either for indoor, outdoor or both types of operating environments - Research papers in which the sensor unit is placed on the white cane - Research papers on virtual white cane and devices that mimic the attributes of the traditional white cane to assist blind users.   Exclusion Criteria:   - Research papers in which the proposed solution is unable to assist blind users through technology - Research papers on assistive devices that do not mimic or extend white cane for navigation - Research papers with less academic significance or with unauthorized publishers or repetitive ideas and thus lack in novelty or originality. | 4 |
| Data collection process | 10 | Describe method of data extraction from reports (e.g., piloted forms, independently, in duplicate) and any processes for obtaining and confirming data from investigators.  **Answer:**  The authors adopted the PRISMA methodology to identify relevant articles. The relevant details were extracted from the selected publications. No user-studies are involved.  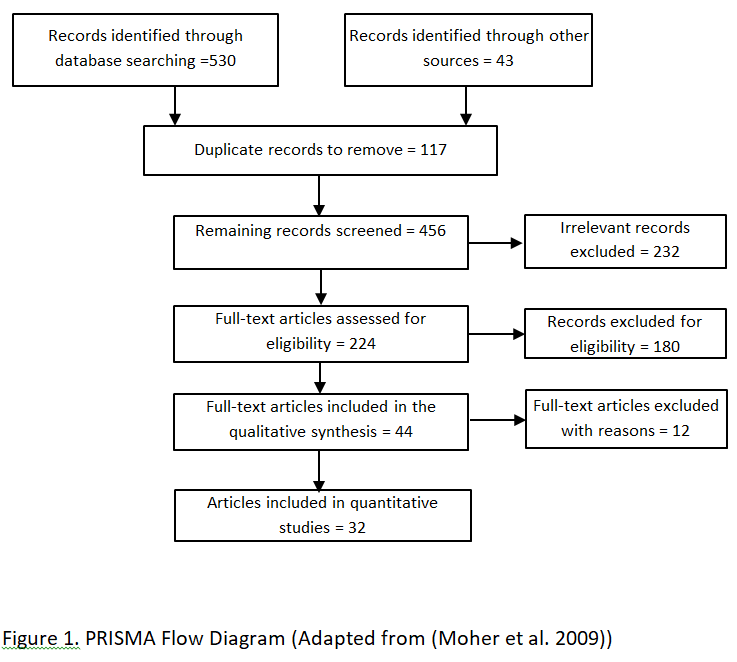 | 4 |
| Data items | 11 | List and define all variables for which data were sought (e.g., PICOS, funding sources) and any assumptions and simplifications made.  **Answer:** Not Applicable |  |
| Risk of bias in individual studies | 12 | Describe methods used for assessing risk of bias of individual studies (including specification of whether this was done at the study or outcome level), and how this information is to be used in any data synthesis.  **Answer:** Not Applicable |  |
| Summary measures | 13 | State the principal summary measures (e.g., risk ratio, difference in means).  **Answer:** Not Applicable |  |
| Synthesis of results | 14 | Describe the methods of handling data and combining results of studies, if done, including measures of consistency (e.g., I^2^) for each meta-analysis.  **Answer:**  Several aspects were considered in synthesis of results. These include   - Operating environments - Use of sensors (single, multiple) - Positioning of sensory unit on white cane - Sensor covering range and area - Mode of operation - Computational devices - Functionality - Localization techniques - User-system interaction (input/output)   The authors used these aspects as the criteria for evaluating and comparing the state-of-the-art technology-assisted white cane solutions designed either for indoor, outdoor or both types of operating environments with the focus on navigation, localization, orientation, and obstacle avoidance. | 5 |

Page 1 of 2

| **Section/topic** | **#** | **Checklist item** | **Reported on page #** |
| --- | --- | --- | --- |
| Risk of bias across studies | 15 | Specify any assessment of risk of bias that may affect the cumulative evidence (e.g., publication bias, selective reporting within studies).  **Answer:** Not Applicable |  |
| Additional analyses | 16 | Describe methods of additional analyses (e.g., sensitivity or subgroup analyses, meta-regression), if done, indicating which were pre-specified.  **Answer:** Not Applicable |  |
| **RESULTS** | | |  |
| Study selection | 17 | Give numbers of studies screened, assessed for eligibility, and included in the review, with reasons for exclusions at each stage, ideally with a flow diagram.  **Answer:**  Out of these 573 studies, 232 were excluded because they unable to match the specified domain of blind assistance through technology. Of the remaining 341, 117 were excluded due to duplication. Full text 224 of studies were considered, 192 papers that did not meet inclusion criteria were excluded. Finally 32 studies were selected (Fig. 1) for the final analysis which match our final inclusion criteria. |  |
| Study characteristics | 18 | For each study, present characteristics for which data were extracted (e.g., study size, PICOS, follow-up period) and provide the citations.  **Answer:**  See Serial No. 14 for more details. Table 1 and 2 should also be considered. | 5 and 9 |
| Risk of bias within studies | 19 | Present data on risk of bias of each study and, if available, any outcome level assessment (see item 12).  **Answer:** Not Applicable |  |
| Results of individual studies | 20 | For all outcomes considered (benefits or harms), present, for each study: (a) simple summary data for each intervention group (b) effect estimates and confidence intervals, ideally with a forest plot.  **Answer:** Not Applicable |  |
| Synthesis of results | 21 | Present results of each meta-analysis done, including confidence intervals and measures of consistency.  **Answer:** Not Applicable |  |
| Risk of bias across studies | 22 | Present results of any assessment of risk of bias across studies (see Item 15).  **Answer:** Not Applicable |  |
| Additional analysis | 23 | Give results of additional analyses, if done (e.g., sensitivity or subgroup analyses, meta-regression [see Item 16]).  **Answer:** Not Applicable |  |
| **DISCUSSION** | | |  |
| Summary of evidence | 24 | Summarize the main findings including the strength of evidence for each main outcome; consider their relevance to key groups (e.g., healthcare providers, users, and policy makers).  **Answer:** Section 4: Results and Discussion, Section 5: Conclusions | 9-14 |
| Limitations | 25 | Discuss limitations at study and outcome level (e.g., risk of bias), and at review-level (e.g., incomplete retrieval of identified research, reporting bias).  **Answer:** Limitations:   - Research papers published in languages other than English are not included. - No coverage research articles related to the issues of power-consumption, stipulated response time, and commercial availability of the technology-assisted white cane. | 14 |
| Conclusions | 26 | Provide a general interpretation of the results in the context of other evidence, and implications for future research.  **Answer:**  See Section 5: Conclusion. A relevant except is included here as an example:  “The white cane is among the most widely used navigation aids for blind people. The advancements in ubiquitous modern technology have introduced numerous opportunities for extending white cane. Researchers have been in the quest to find out ways of extending white cane existing technology. However, for a more reliable extension, the design should be user-centric characteristics. It should be portable, reliable, trust-worthy, lightweight, less costly, less power hungry, and require minimal training. In this regard, smartphones, which are the ubiquitous and general-purpose portable devices, should be considered to exploit its capabilities in making technology-assisted white cane smarter and reliable.” | 12-14 |
| **FUNDING** | | |  |
| Funding | 27 | Describe sources of funding for the systematic review and other support (e.g., supply of data); role of funders for the systematic review.  **Answer:**  Not Applicable |  |

*From:*  Moher D, Liberati A, Tetzlaff J, Altman DG, The PRISMA Group (2009). Preferred Reporting Items for Systematic Reviews and Meta-Analyses: The PRISMA Statement. PLoS Med 6(7): e1000097. doi:10.1371/journal.pmed1000097

For more information, visit: **www.prisma-statement.org**.

Page 2 of 2
